# Effect of Digested Selected Food Items on Markers of Oxidative Stress and Inflammation in a Caco-2-Based Human Gut Epithelial Model

**DOI:** 10.3390/antiox13020150

**Published:** 2024-01-25

**Authors:** Farhad Vahid, Pit Krischler, Bernadette Leners, Torsten Bohn

**Affiliations:** Nutrition and Health Research Group, Department of Precision Health, Luxembourg Institute of Health, L-1445 Strassen, Luxembourg; farhad.vahid@lih.lu (F.V.); pit.krischler@education.lu (P.K.); bernadette.leners@lih.lu (B.L.)

**Keywords:** reactive oxygen species, cytotoxicity, antioxidant capacity, interleukins, peroxidation, INFOGEST

## Abstract

The human gut epithelium presents a crucial interface between ingested food items and the host. Understanding how different food items influence oxidative stress and inflammation in the gut is of great importance. This study assessed the impact of various digested food items on oxidative stress, inflammation, and DNA/RNA damage in human gut epithelial cells. Differentiated Caco-2 cells were exposed to food items and their combinations (n = 22) selected from a previous study, including sausage, white chocolate, soda, coffee, orange juice, and curcumin. Following stimulation with TNF-α/IFN-1β/LPS and H_2_O_2_ for 4 h, the cells were exposed to digested food items or appropriate controls (empty digesta and medium) for a further 16 h. Cell viability, antioxidant capacity (ABTS, FRAP), IL-6, IL-8, F2-isoprostanes, lipid peroxidation (MDA), and DNA/RNA oxidative damage were assessed (3 independent triplicates). The ABTS assay revealed that cells treated with “white chocolate” and “sausage + coffee” exhibited significantly reduced antioxidant capacity compared to stimulated control cells (ABTS = 52.3%, 54.8%, respectively, *p* < 0.05). Similar results were observed for FRAP (sausage = 34.9%; white chocolate + sausage = 35.1%). IL-6 levels increased in cells treated with “white chocolate + sausage” digesta (by 101%, *p* < 0.05). Moreover, MDA levels were significantly elevated in cells treated with digested “sausage” or sausage in combination with other food items. DNA/RNA oxidative damage was found to be higher in digesta containing sausage or white chocolate (up to 550%, *p* < 0.05) compared to stimulated control cells. This investigation provides insights into how different food items may affect gut health and underscores the complex interplay between food components and the epithelium at this critical interface of absorption.

## 1. Introduction

The human body and its tissues, under normal conditions, are constantly in contact with molecular oxygen (O_2_). Whereas most of the oxygen consumed by the human body is used for energy generation, an estimated 5% of the oxygen is involved in the formation of reactive oxygen species (ROS), especially including hydrogen peroxide (H_2_O_2_), superoxide (O_2_^•−^), and hydroxyl radicals (^•^OH), as well as singlet oxygen (^1^O_2_) [1]. Low ROS concentrations at the physiological level are indispensable for human health, considering their key roles in regulating intracellular processes [2]. It is proven that low concentrations of ROS are required in multiple cellular signaling pathways, such as protein kinase activation and gene transcription, and are involved in coordinated cell motility, cytokine regulation, and wound healing [2,3]. In the case of a disturbance of the equilibrium between the formation and the neutralization of ROS, however, oxidative stress can lead to a breakdown of cellular and membrane functions due to their tendency to oxidize proteins, lipids, and carbohydrates, as well as nucleic acids [4]. Further studies have underlined the implications of ROS in pathological processes, such as inflammation [5,6]. This includes negative effects of ROS in the gut at the interface between the lumen and the intestinal tissue [7]. Long-term exposure and disproportionate generation of ROS in the gut can lead to the development and progression of various diseases, including enteric infections, inflammatory bowel disease (IBD) [8], and ischemic intestinal injury, as well as different colorectal cancers [2,9,10].

In order to counter harmful ROS concentrations, aerobic organisms rely not only on endogenous enzymatic (e.g., superoxide dismutase or catalase) and non-enzymatic antioxidants (e.g., glutathione) but also on exogenous antioxidants, such as vitamin C or E, as well as certain phytochemicals such as polyphenols or carotenoids [8,11]. These antioxidants are present in various food items, especially plant-based food items including coffee beans, fruits, vegetables, and their products, such as orange juice or coffee. It is also noted that, in addition to direct quenching effects, the antioxidant activity can also be transferred via more indirect effects, such as by impacting cellular signaling cascades involving the transcription factor Nrf2, important for the body’s own antioxidant defense system [12].

On the other hand, especially in the case of processed food, the contents and the processing techniques can reduce antioxidant capacities or even increase pro-oxidant levels, as well as the pro-inflammatory potential of such foods [13]. For example, sugar-rich beverages such as sodas have been reported to increase ROS production in the short term in animal experiments [14], downregulating the body’s own antioxidant defense mechanisms, while the consumption of meat can produce lipid peroxides [15]. In a previous study of ours, we have shown that lipid-rich food matrices such as white chocolate and sausage indeed result in the production of malondialdehyde (MDA), a downstream product of peroxidation during digestion, which, however, could be quenched by simultaneous ingestion of orange juice [16].

However, little is known about the further effects of food combinations regarding their pro-or antioxidant effect on the gut epithelium. While it is well-established that oxidative stress and inflammation can have adverse effects on the human intestine and are associated with various diseases, such as IBD [17,18], there is a need to investigate further the specific effects of selected food items on oxidative stress and inflammation and associated biomarkers in the human gut epithelium. Specifically, there is a research gap in understanding how the intake of different food items, including those rich in exogenous antioxidants and phytochemicals such as polyphenols, as well as foods with potential pro-oxidant effects such as processed foods, and also their combinations, may modulate these biomarkers in the gut epithelium.

This investigation aimed to investigate the impact of selected frequently consumed food items and their combinations on markers of oxidative stress and inflammation in the human gut epithelium. We hypothesized that consuming food items rich in exogenous antioxidants and phytochemicals would lead to a reduction in oxidative stress markers and inflammatory responses in the human gut epithelium. Conversely, we expected processed foods to have the opposite effect, increasing the pro-oxidant and pro-inflammatory potential.

## 2. Materials and Methods

### 2.1. Food Items and Rationale of Selection

In the present study, different food items and their combinations (Table 1) were tested following simulated gastro-intestinal digestion on a model of the human gut epithelium (Caco-2) in order to investigate their pro-and antioxidant effects, as well as their inflammatory effects. Food items were selected based on a previous study [16] covering a broad range of frequently consumed food items regarding their effect on oxidative stress following gastro-intestinal digestion in the digestive fluids.

### 2.2. Chemicals

Pepsin (>250 units/mg activity, product No. P7000), porcine bile extract (product no. B8631), and pancreatin (4xUSP specifications, product No. P1750), as well as hydrogen peroxide (3%, suitable for microbiology) and L-ascorbic acid (vitamin C, 99% purity) were purchased from Sigma Aldrich (St. Louis, MO, USA). Curcumin, methanol, and hydrochloric acid were acquired from Apollo Scientific (VWR, Leuven, Belgium). Dulbecco’s modified eagle medium (1×) + GlutaMax and ethyl acetate were purchased from Fisher Scientific (Merebeke, Belgium), as was the heat-inactivated fetal bovine serum. The non-essential amino acids mixture (NEAA) (100×), the pen/strep mixture (10,000 U penicillin/mL, 10,000 U streptomycin/mL), as well as the DPBS solution without calcium or magnesium, were purchased from LONZA BioWhittaker (Westburg, Belgium). IL-1β was from Abcam (Waltham, Boston, FL, USA). Unless stated otherwise, all other used chemicals were purchased from Sigma-Aldrich.

### 2.3. Simulated Gastro-Intestinal (GI) Digestion

The digestion protocol was performed according to the INFOGEST consensus model for static digestion [20], specifying all details of digestive conditions. The oral phase involving simulated salivary fluids (SSFs) was removed for practical reasons, as food items investigated were either liquid or low in starch due to the short exposure, similarly to work carried out previously [21].

In total, 11 food items were digested, either single or in their combination (Table 1). All food items were purchased from a local supermarket (Delhaize, Strassen, Luxembourg). For each condition, 1.04 mL of pepsin solution (25,000 U/mL) was added to the tubes, followed by the addition of 4.16 mL of simulated gastric fluid (SGF) solution, as well as 32.5 µL of CaCl_2_ (0.03 M). After mixing, the pH value of the samples was adjusted to pH 3 ± 0.4, using 1 M HCl. Next, the volume of the samples was brought up to a total of 13 mL with pure water and the tubes were closed tightly and sealed in a plastic bag. The samples were incubated for 2 h in a shaking water bath at 37 °C and 100 rpm. Special attention was given to ensure that the falcon tubes were completely submerged and placed horizontally in the direction of the shaking movement. After one hour of incubation, the pH was checked and readjusted where needed.

After gastric incubation, 2.6 mL of 2000 U/mL pancreatin and 68.8 mg/mL bile extract solution were added to the samples, followed by 7.8 mL of simulated intestinal fluid (SIF) solution, as well as 260 µL of CaCl_2_ (0.03 M). The pH was adjusted to 7 ± 0.4, and the volume of the samples was brought up to a total of 26 mL with pure water before incubation for another 2 h. Following intestinal digestion, the digested samples were vortexed, and 15 mL of digesta from 3 independent digestion experiments were pooled and stored at −80 °C until further use for the cell culture experiments.

### 2.4. Cell Culture

Caco-2 cells (adherent epithelial colon tissue cells isolated from a male with colorectal adenocarcinoma, passage 68) were provided by ATCC (ATCC Number: HTB-37, LGC Standards, Molsheim, France).

The cell culture medium used to culture the cells was DMEM + glutamax, where 10% FBS, 1% PenStrep, and 1% NEAA was added.

Cells were grown in T75 flasks and split about twice per week whenever they reached 90% confluency (1:8).

For cellular experiments, Caco-2 cells were seeded in 6-well culture plates (ThermoScientific Nunclon™ Delta Surface, Gent, Belgium) at a density of 50,000 cells per cm^2^ and let to differentiate at 37 °C and 5% ambient CO_2_ for a period of 2 weeks, during which the growth medium was changed every 2 days. After differentiation, the effects of 26 different treatments were studied (Table 2).

A stimuli solution was prepared to produce incubation concentrations of 100 ng mL^−1^ TNF-α, 25 ng mL^−1^ IL1β, 10 µg/mL^−1^ LPS, and 0.4 mmol L^−1^ H_2_O_2_ in a cell culture medium. The prepared stimuli solution was sterilized by filtration (VWR Syringe Filter Nylon 0.22 µm). The stimuli types and concentrations were selected based on earlier trials [22].

For the exposure group “Blank + digesta”, the digesta of the different food items were diluted 1:8 in the cell culture medium, based on previous studies indicating that this suffices to reduce the negative impact of bile salts and enzymes present in the digesta on Caco-2 cells [21]. For the exposure group “Blank + stimuli + digesta”, the digesta of the different food items were diluted 1:8 in cell culture medium containing added stimuli. Both of the digesta solutions were sterilized by filtration. The cell culture medium was removed from the culture wells at the start of the cell treatment. For unstimulated cells, 2 mL of fresh culture medium replaced the old culture medium. Otherwise, the cell culture medium was replaced with 2 mL of stimuli-containing medium. After incubation (4 h), the culture medium from the cells of the exposure group “Blank + digesta” was removed and replaced by 1.8 mL of the diluted digesta in the culture medium. For the cells of the exposure group “Blank + stimuli + digesta”, the stimuli solution was removed from the wells and replaced by 1.8 mL of diluted digesta containing the added stimuli. Next, the 6-well plates were incubated for a further 16 h in unchanged culture conditions to simulate intake from repeated meals rather than from a single intake, similar to experiments performed in previous studies [23].

Cells were then harvested from the 6-well plates by scraping. The detached cells and the supernatants were transferred into 2 mL centrifuge tubes. The cells were separated from the culture medium by centrifuging the tubes at 300× *g* for 3 min (Eppendorf Centrifuge 5810R, Eppendorf, Nijmegen, The Netherlands). The supernatant was transferred to new 2 mL centrifuge tubes, and aliquots were stored at −80 °C; the pellet was re-suspended by adding 600 µL of sterile water, followed by vortexing and an incubation of 5 min at RT. Next, the samples were sonicated for 10 min at RT to complete lysis before storage at −80 °C. On the following day, the lysed cells were thawed, vortexed, and sonicated for 5 min, centrifuged at 16,000× *g* for 5 min, and the supernatant was aliquoted. Aliquots of the cellular supernatants were kept frozen at −80 °C under argon until analysis.

### 2.5. LDH Cytotoxicity Test

LDH cytotoxicity assays were provided by Roche (art. No. 11644793001, Sigma Aldrich) and conducted according to the manufacturer’s manual. To evaluate the total cell viability of the treated cells, one positive and one negative control well, both containing untreated, unstimulated cells, were measured together within the main cellular exposure experiments ± 10 min prior to harvesting the treated cells. Then, 2% of Triton X-100 was added to the positive control well. Cell viability was expressed as a mean (%) compared to cells without digesta treatment but treated with Triton X-100 (2% in culture medium), which was set to 100%. Next, 100 µL of the harvested cell culture mediums, as well as of the positive and negative controls, were added to the wells of a 96-well plate (Greiner Bio-one microplate, 96 well, PS, F-Bottom, clear, Ref. 655101). Then, the reaction mixture (100 µL) was added to each well. After 30 min of incubation, the absorbance was read at 490 nm (POLARstar OPTIMA BMG Labtech, Ortenberg, Germany). After blank subtraction, the cytotoxicity was calculated relatively to the negative control (all cells alive) and the positive control (all cells dead).

### 2.6. Extraction and Quantification of F2-Isoprostanes

For the F2-isoprostane extraction, harvested cell material was used. Upon thawing the frozen cell samples, 1 volume of 40% KOH in methanol was added to the Eppendorf tubes proportionally to the volume of cell lysates. The samples were then vortexed and incubated for 1 h in a block heater at 50 °C. After incubation, the acidity of the lysates was adjusted to pH ± 4–5 by adding formic acid (98%) and the content was transferred to 15 mL Falcon tubes. After adding 2 volumes of ethyl acetate and vortexing, the Falcon tubes were centrifuged at 2000× *g* for 10 min at RT, and the upper phase (containing the isoprostanes) was transferred to a new 15 mL Falcon tube. The extraction was repeated with 2 mL ethyl acetate. Next, the combined ethyl acetate phase was evaporated at 40 °C under a stream of nitrogen (Biotage TurboVap LV Concentrator Workstation, Uppsala, Sweden). The dried extracts were stored at −80 °C under argon.

Quantification of F2-isoprostanes was achieved by an ELISA kit (Cayman, art. no. 516351, purchased from Sanbio, Uden, The Netherlands) according to the manufacturer’s protocol. The dried F2-isoprostane extracts were dissolved by adding 200 µL of ELISA buffer to the 15 mL tubes containing the samples. The ELISA test was performed on 96-well plates (pre-coated well strips provided in the kit). The standards required were prepared according to Supplementary Table S1. After the final incubation, the plate was read at a wavelength of 405 nm (POLARstar OPTIMA). After the blank subtraction, the F2-isoprostane concentrations were determined using a logit-transformed standard curve.

### 2.7. ABTS Assay

The ABTS assay was conducted on lysed cell material, similar to what was explained earlier [22]. Briefly, an ascorbic acid stock solution of 1 g ascorbic acid/100 mL was prepared. Based on this stock solution, standards were prepared according to Supplementary Table S2. Next, a radical solution containing 10 mg of AAPH and 50 mg of ABTS per 50 mL PBS buffer was prepared. The radical solution was checked to be at 7.4 pH before incubation in a water bath at 73 °C for 40 min. After cooling down, the solution was centrifuged at 2000× *g* for 4 min, and the supernatant was recovered into a fresh tube.

The absorption of the radical solution against the blank (PBS) was measured at 750 nm in a POLARstar plate reader and was checked to read 1.0 ± 0.2. Next, 20 µL of the samples, MilliQ water (blanks), and standards were mixed with 980 µL of the ABTS radical solution and incubated for 15 min in a heating block at 37 °C. After incubation and sonication, the tubes were centrifuged at 8000× *g* for 3 min, and 300 µL of the supernatants were transferred in a 96-well plate and measured in the plate reader at 750 nm.

### 2.8. FRAP Assay

The assay was conducted on lysed cell material, as described earlier [22]. In short, 100 mL of an acetate buffer was prepared (65.3 mg of sodium acetate and 570 µL acetic acid 96% in 100 mL, pH 3.6). TPTZ (15.6 mg) was suspended in 5 mL of 40 mM HCl. In addition, an iron (III) chloride solution was prepared by dissolving 27.03 mg of iron(III)chloride (hexahydrate) in 5 mL of MilliQ water. A standard iron(II)chloride stock solution was prepared by dissolving 38.02 mg in 15 mL of Milli-Q water. A standard series was prepared according to Supplementary Table S3.

The FRAP reagent was prepared by mixing 25 mL of the acetate buffer with 2.5 mL of the TPTZ-HCl solution and 2.5 mL of the iron(III)chloride (hexahydrate) solution. The FRAP reagent was heated in a water bath at 37 °C for 15 min. Next, 100 µL of standards were mixed with 750 µL of the cooled FRAP reagent and incubated for 30 min (room temperature). The tubes were then centrifuged at 2000× *g* for 3 min, and 300 µL of the supernatant was pipetted into the wells of a transparent 96-well Greiner plate, and the absorbance was read at 595, as well as at 465 and 750 nm for measuring the background absorption (SpecraMax M2, Molecular Devices, San Jose, CA, USA) within 1 h.

### 2.9. IL-6 and IL-8 Assays

The IL-6 ELISA assay was performed on cell culture supernatant using a kit from Cayman Chemicals (art. No. 501030). For IL-8, an ELISA kit from Invitrogen (article No. 88-8086) was used, following the manufacturer’s protocol. The standards were prepared according to the Supplementary Table S4a,b. The absorbance of the wells was read at 450 nm (POLARstar plate reader) for IL-6 and IL-8.

### 2.10. MDA Assay

The MDA assay was performed on lysed cell material, as described earlier [16], using a Cayman kit (art. No. 700870), following, in general, the manufacturer’s protocol. MDA standards were prepared as detailed in (Supplementary Table S5. Final solutions (300 µL) were measured in a black 96-well plate (Greiner Bio-one microplate F-bottom, black, Fluotrac, high binding) using fluorescence, employing an excitation wavelength of 525 nm and an emission wavelength of 565 nm (SpectraMax M2).

### 2.11. DNA/RNA Oxidative Damage

DNA/RNA oxidative damage was assessed by the DNA/RNA oxidative damage ELISA kit (Cayman Chemicals, art. No. 589320), following the manufacturer’s protocol. The ELISA assay was performed on lysed cell material. A standard dilution series was prepared from the diluted standard solution according to Supplementary Table S6. The final developed plate was read at a wavelength of 405 nm (POLARSTAR plate reader); standard curves were prepared by logit-transformation.

### 2.12. Data Treatment and Statistical Approach

Raw values were corrected by subtracting the respective blank values. A Grubbs’ test was performed to screen for outliers, which were replaced by mean values. Normality of distribution and equality of variance were tested by Q-Q plots and box-plots, respectively. In all analyses, the log-transformed values were used. Values were expressed within one analysis compared to the daily control (cells with media and stimuli and empty digesta), which was set to100%. Multivariate analysis followed by Dunnett’s post hoc test was used to study the effect of food items and their combinations on log-transformed values of FRAP, ABTS, MDA, IL-6, IL-8, DNA/RNA damage, F2-isoprostanes, and cytotoxicity versus the controls. Spearman rank correlations were employed to measure the strength and direction of monotonic association between inflammatory-, oxidative stress-, DNA/RNA oxidative damage-, and cytotoxicity variables. Data were analyzed with SPSS (IBM, Chicago, IL, USA) version 25.0. A *p*-value of <0.05 (2-sided) was considered statistically significant.

## 3. Results

### 3.1. Multivariate Analysis

The *p*-value for the multivariate test was highly significant (*p* < 0.001), suggesting that the food items had a significant overall effect on the various endpoints measured. Likewise, the food group (including blank and stimuli conditions) significantly impacted each of the following studied parameters, *p* < 0.001.

#### 3.1.1. ABTS

**Figure 1:**
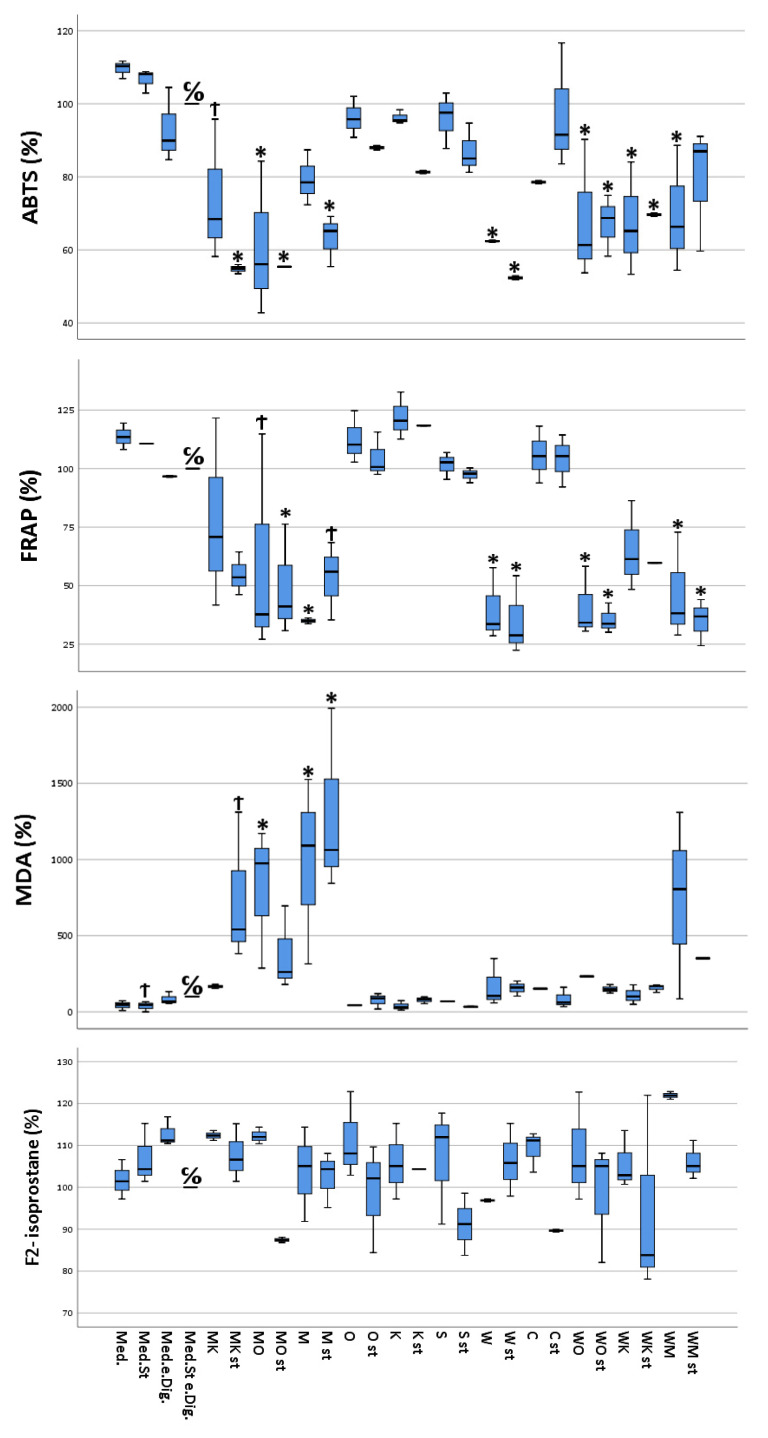
Multivariate analysis using Dunnett’s test comparing antioxidant and pro-oxidant (ABTS, MDA, FRAP, and F2-isoprostane) activity of selected food items and their combinations following simulated gastro-intestinal digestion in differentiated Caco-2 cell monolayers. All values were measured following the lysis of Caco-2 cells. All values are expressed as percentages compared to their controls (medium plus stimuli and “empty digesta” (digesta without any food item)). Abbreviations: med = medium (blank); med.st. = medium + stimuli; med.e.dig = medium + empty digesta; med.st.e.dig. = medium + stimuli + empty digesta; MK = sausage (meat) + coffee; MK st = sausage (meat) + coffee + stimuli; MO = sausage (meat) + orange juice; MO st = sausage (meat) + orange juice + stimuli; M = sausage (meat); M st = sausage (meat) + stimuli; O = orange juice; O st = orange juice + stimuli; K = coffee; K st = coffee + stimuli; S = soda; S st = soda + stimuli; W = white chocolate; W st = white chocolate + stimuli; C = curcumin; C st = curcumin + stimuli; WO = white chocolate + orange juice; WO st = white chocolate + orange juice + stimuli; WK = white chocolate + coffee; WK st = white chocolate + coffee + stimuli; WM = white chocolate + sausage (meat); WM st = white chocolate + sausage (meat) + stimuli. C/O = control group; Ϯ = trend (0.05 > *p*-value > 0.1); * The mean difference was significant at the 0.05 level (Dunnett’s post hoc test). Tests of between-subjects effects indicated statistically significant differences.

The results of the ABTS test on the different Caco-2 samples showed antioxidant capacities ranging between 0.06 mg vitamin C equivalent/mL (stimulated cells treated with “sausage + coffee” digesta) and 0.11 mg vitamin C equivalent/mL (unstimulated cells, without treatment). It is apparent that the cells treated with digesta containing either “sausage”, “white chocolate”, or a combination of these food items, in which either one was present, showed an overall reduced antioxidant capacity compared to cells treated with other digesta or without digesta. Furthermore, in the majority of the analyzed cell samples, for the same cellular treatment, a lower antioxidant capacity was observed for the stimulated cells compared to the unstimulated counterparts (Figure 1).

#### 3.1.2. FRAP

FRAP values ranged from a minimum of 11.4 mg iron II chloride equivalent/L (stimulated cells, treated with “white chocolate” + sausage digesta) to a maximum of 36.9 mg iron II chloride equivalent/L (unstimulated cells, treated with “coffee” digesta). As for ABTS, the cells treated with digested food item combinations containing either “sausage” or “white chocolate”, showed a significantly reduced antioxidant capacity compared to the Caco-2 cells treated with either the remaining food digesta or cells without digesta. In addition, the stimulated cells exhibited a reduced antioxidant capacity compared to their untreated counterparts (Figure 1).

#### 3.1.3. MDA

MDA concentrations ranged between 0.09 µM MDA (ca. 6.5 µg/L, stimulated and untreated cells) and 3.88 µM MDA (ca. 280 µg/L, stimulated cells treated with “sausage” digesta). The MDA concentrations were significantly increased for the cells treated with either digested sausage alone or sausage in combination with other food items. Furthermore, no significant correlation between the state of stimulation of the cells and the measured MDA concentrations could be observed (Figure 1).

#### 3.1.4. F2-Isoprostane Analysis

Concentrations of F2-isoprostanes showed large variability between the samples of the biological triplicate, with percentages ranging from 87.4% (sausage + orange juice) to 121.9% (“white chocolate” + sausage) (Figure 1).

#### 3.1.5. Cytotoxicity

**Figure 2:**
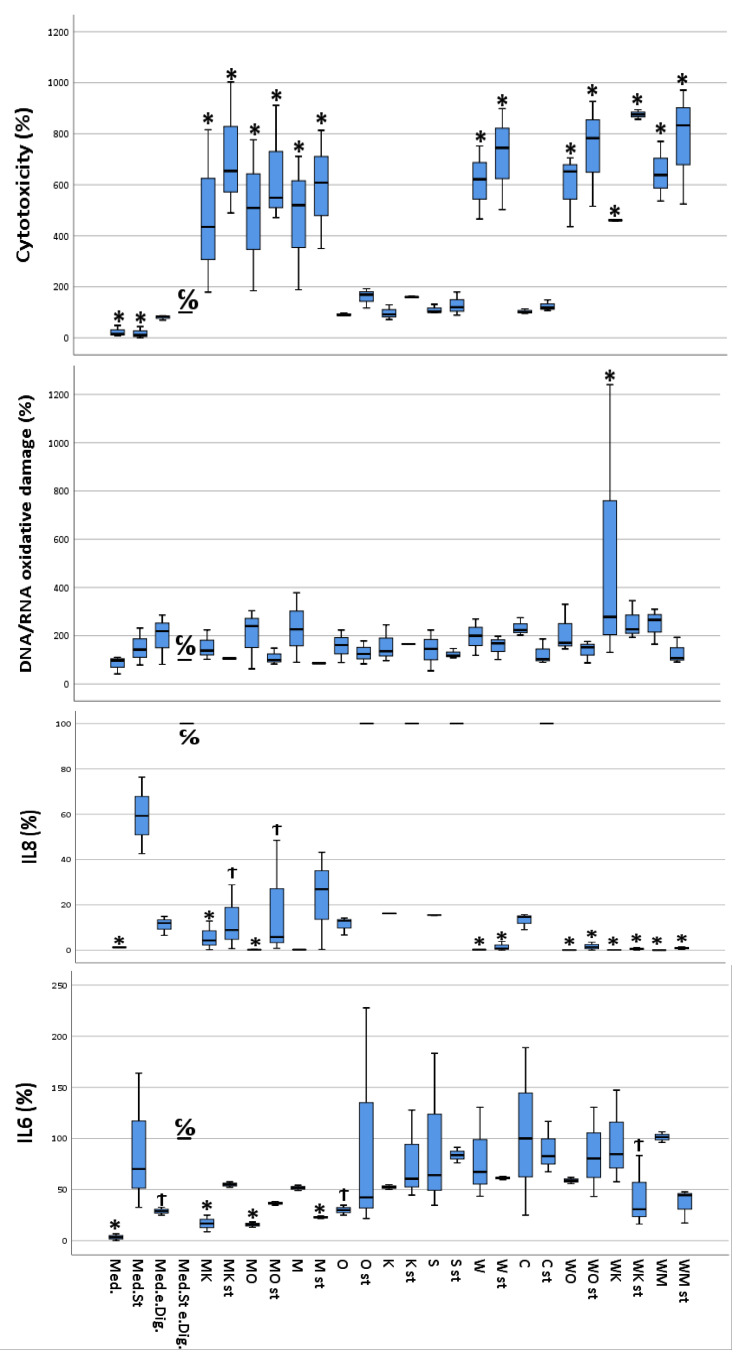
Multivariate analysis using Dunnett’s test comparing cytotoxicity, DNA/RNA oxidative damage, and inflammatory activity (IL-6 and IL-8) of selected food items and their combinations following simulated gastro-intestinal digestion in differentiated Caco-2 cell monolayers. IL-6 and IL-8 were measured in the cellular supernatants, DNA/RNA in the lysed cells, and cytotoxicity as described in materials and methods. All values were measured following the lysis of Caco-2 cells. All values are expressed as percentages compared to their controls (medium plus stimuli and “empty digesta” (digesta without any food item). Abbreviations: med = medium (blank); med.st. = medium + stimuli; med.e.dig = medium + empty digesta; med.st.e.dig. = medium + stimuli + empty digesta; MK = sausage (meat) + coffee; MK st = sausage (meat) + coffee + stimuli; MO = sausage (meat) + orange juice; MO st = sausage (meat) + orange juice + stimuli; M = sausage (meat); M st = sausage (meat) + stimuli; O = orange juice; O st = orange juice + stimuli; K = coffee; K st = coffee + stimuli; S = soda; S st = soda + stimuli; W = white chocolate; W st = white chocolate + stimuli; C = curcumin; C st = curcumin + stimuli; WO = white chocolate + orange juice; WO st = white chocolate + orange juice + stimuli; WK = white chocolate + coffee; WK st = white chocolate + coffee + stimuli; WM = white chocolate + sausage (meat); WM st = white chocolate + sausage (meat) + stimuli. C/O = control group; Ϯ = trend (0.05 > *p*-value > 0.1); * The mean difference was significant at the 0.05 level (Dunnett’s post hoc test). Tests of between-subject effects were statistically significant except for DNA/RNA oxidative damage (*p* = 0.089).

Depending on the different states of stimulation and treatment by the different digested food items, the cultured CacCo-2 cells exhibited different cellular viabilities. The calculated cytotoxicity ranged between 1.8% (calculated for stimulated cells without digesta treatment) and 87.5% (calculated for stimulated cells treated with the “sausage + white chocolate” digesta). Furthermore, a slight difference in cytotoxicity was observed between the unstimulated cells treated with a certain digesta and their stimulated counterparts, with the stimulated cells showing increased cytotoxicity (Figure 2).

#### 3.1.6. IL-6

The data from the IL-6 quantification performed on the differently treated Caco-2 cells ranged from 4 pg IL-6/mL (unstimulated and untreated cells) to a 30 pg IL-6/mL (unstimulated cells, treated with the “white chocolate + sausage” digesta). Interestingly, the stimulated cells treated with the digesta-containing soda released a similar concentration (30 pg IL-6/mL) of IL-6 into the cell culture medium as the cells treated with the “white chocolate + sausage” digesta. Furthermore, in the majority of the cells treated with the same digesta, the cells that had been stimulated exhibited a higher IL-6 concentration compared to their unstimulated counterparts (Figure 2).

#### 3.1.7. IL-8

IL-8 concentrations in the cellular supernatants ranged from 1.29 pg/mL (unstimulated cells treated with media) to 6250 pg/mL or higher (stimulated cells treated with empty digesta, highest calibration point). The control samples already reached the maximum absorption response (medium plus stimuli plus empty digesta); thus, higher responses could not be further differentiated (Figure 1). Interestingly, complex food matrices such as white chocolate and meat showed lower concentrations of IL-8 in cellular supernatants compared to less complex matrices, i.e., orange juice, coffee, or curcumin. Furthermore, in the majority of the cells treated with the same digesta, the cells which were stimulated exhibited a much higher IL-8 concentration compared to their unstimulated counterparts (Figure 2).

#### 3.1.8. DNA/RNA Oxidative Damage

The measured DNA/RNA oxidative damage of the cells ranged between 87.6 ng guanine species/mL (unstimulated and untreated cells) and 280.5 ng guanine species/mL (unstimulated cells treated with “curcumin” digesta). For most of the cells treated with the same digesta, the stimulated cells showed increased DNA/RNA oxidative damage compared to their unstimulated counterparts (Figure 2).

### 3.2. Correlation Analyses

**Figure 3:**
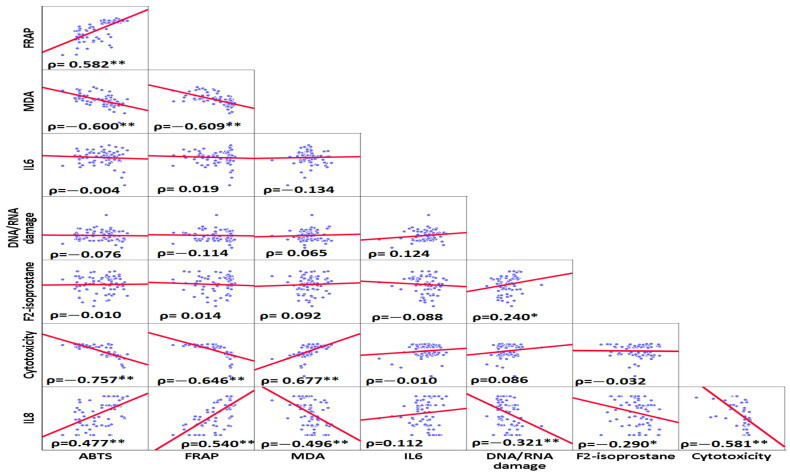
Spearman ranks correlations between inflammatory and oxidative stress, DNA/RNA oxidative damage, and cytotoxicity variables. The highest positive correlation was found between MDA and cytotoxicity. The highest negative correlation was observed between cytotoxicity and ABTS. * Correlation was significant at the <0.05 level (2-tailed) ** Correlation was significant at the <0.01 level (2-tailed).

The correlation analyses are shown in Figure 3. Based on the Spearman correlation matrix, the highest positive correlation was observed between MDA and cytotoxicity (ρ = 0.677, *p*-values < 0.001), and the highest negative correlation was found between ABTS and cytotoxicity (ρ = −0.757, *p*-values < 0.001) (Figure 3).

## 4. Discussion

The present study investigated oxidative stress and inflammatory markers of digested food items and their combinations at the interface of the gut and the host, i.e., in a model of the small intestinal epithelium, where most macro- and micronutrients are absorbed [24]. Previous studies have suggested that ROS and pro-oxidant food compounds could especially negatively impact the gut epithelium, as ROS levels post-digestion could be high [9,16], i.e., higher than in native food items or in other body compartments that are under tight homeostatic ROS control, though surprisingly little knowledge exists in this area. As ROS is tightly linked to inflammation, food items rich in ROS or increasing ROS during digestion could also increase systemic inflammation [25]. We hypothesized that food items and their combinations with diverging amounts of antioxidant profiles would result in similar diverging levels of markers of oxidative stress and inflammation in the gut epithelium due to direct quenching effects of antioxidants [26], the pro-oxidant properties of some food constituents such as lipid peroxides [27,28,29], or due to the impact of food constituents on transcription factors such as Nrf2 [8,30,31].

As a first measure of cumulative cellular damage, the measured cytotoxicity of the differently treated Caco-2 cells significantly increased in cells treated with digesta, especially those containing “sausage” and/or “white chocolate” compared to control conditions or those exposed to orange juice, coffee, curcuma, and also soda beverages. The added stimulants (TNF-α, LPS, IL-1β, and H_2_O_2_), chosen in accordance with earlier studies [32] appeared to only slightly augment cytotoxic effects in the presence of digesta. A previous study of ours has already suggested that the presence of high levels of pro-oxidants such as MDA in white chocolate and sausage [16] and H_2_O_2_ (present in our media at 400 µmol/L) can increase oxidative stress on the cellular level, already at the nanomolar level [33,34]. Likewise, the cytotoxic effects of cytokines such as TNF-α are well-recognized [35]. It is also known that fatty acids such as palmitic acid, potentially released during gastro-intestinal digestion from food items including chocolate or meat, could induce inflammation in the gut epithelium [36]. These findings are in line with other cellular studies finding elevated levels of cytotoxicity in Caco-2 cells upon exposure to lipids, especially when combined with emulsions and medium-chain fatty acids that are also both expected to be present in chocolate and sausage [37].

The cytotoxicity findings are mirrored in the results of the antioxidant capacity of the cells, i.e., ABTS and FRAP, showing a decreased capacity of cells exposed to white chocolate and sausage, reducing antioxidant capacity down to 40% of its original level, underlining the possible close relation between cell mortality and oxidative stress in Caco-2 cells [38]. Aside from the possible higher levels of MDA originating from lipid digestion and peroxidation, we can only speculate about the nature of the decreased antioxidant capacity. A previous study has emphasized the effects of high-fat diets on increased oxidative stress [39] in the gut mucosa of a mouse model, which was linked to an upregulation of the NADPH oxidase enzyme, lowering the overall cellular antioxidant capacity. The fact that sausage was also rich in nitrite and ascorbic acid and potential antioxidants [40], as well as releasing peptides upon digestion that could have antioxidant properties [41], interestingly did not appear to convey any positive effects in the cell model, contrary to high ABTS levels previously observed in sausage-containing digesta [16], perhaps due to previous degradation of ascorbic acid and nitrite during digestion and/or limited cellular uptake of these antioxidants. In the future, it would also be of interest to study nitrosamines, as these potentially cancerogenous compounds may be generated by interactions between nitrites and proteins present in meat during digestion, i.e., in acidic conditions [42]. Originating nitrogen species such as nitric oxide could also increase nitric stress, even though some compounds such as NO may also have vessel beneficial effects [43].

It should be noted that from all the cell samples that were analyzed by the ABTS and FRAP assays, only the cell samples treated with “coffee” and, to a lesser degree, orange juice exhibited an antioxidant effect, which was equal to, or slightly (but not significantly) higher than, the cells treated with digesta without added food items. The latter result may indicate the high antioxidant properties of brewed “coffee”. Due to the high number of phytochemical components, including polyphenols and Maillard products originating during roasting [44], which both have the capacity to scavenge free radicals, provide reducing activity, as well as acting as chelators for pro-oxidant metal ions [45]. Interestingly, soda did not reduce antioxidant activity in ABTS or FRAP significantly. Despite the fact that sugar can cause acute oxidative stress upon its metabolism, e.g., by NADPH oxidase pathway or mitochondrial oxidation, but also uric acid production [46], such reactions may take more time than the chosen 16 h exposure, reflecting a more acute exposure. In a study exposing Caco-2 cells for 1 week to high glucose concentrations, an increased intracellular ROS formation was found [47].

Regarding IL-6, of all the samples analyzed, the unstimulated cells and those treated with digesta devoid of food items released the lowest levels of IL-6 into the culture medium, confirming that both the added stimuli and the different digesta could influence the inflammatory state of the cells. Compared to the non-stimulated control cells treated with the empty digesta, the addition of orange juice and coffee did not seem to have any negative effect, while the addition of white chocolate, sausage, and also, soda showed a tendency (but not significantly) toward increased inflammatory reactions. Similar effects regarding a slight reduction of IL-6 secretion in a Caco-2 co-culture cell model with antioxidant-rich kale was found earlier [23]. However, adding food items did not further augment the inflammatory state compared to the already stimulated cells in the present investigation. The inflammatory effect of the digested soda and perhaps chocolate on the cells could be explained by its high glucose content, which has been shown to increase epithelial permeability and fostering inflammation in Caco-2 cells [48] The high inflammatory effect of the digesta-containing sausage and possibly white chocolate may be explained by the potential inflammatory effect of pro-oxidative reactions caused by oxidized lipids in meat and chocolate [47,49]. Negative effects of oxidized lipids on markers of inflammation and oxidative stress, related to increased NF-ĸB expression were shown in earlier in vitro and in vivo animal models [50]. Contrarily to IL-6, no negative effects of adding sausage and white chocolate were apparent for IL-8 compared to respective unstimulated controls, rather lower levels of IL-8 for matrices containing sausage and white chocolate were observed, for reasons unknown, even unspecific binding of IL-8 to proteins present in the digesta cannot be excluded.

In line with these findings, the malondialdehyde (MDA) assay results showed a considerable increase in lipid peroxidation activity in the cell samples treated with digesta containing sausage. The cell samples treated with digesta containing “white chocolate” showed the second-highest MDA concentrations of all analyzed samples. However, the considerably high peak of MDA content of the samples treated with sausage-containing digesta appears to not be proportional to the decrease in the antioxidant potential of those samples. It could thus be hypothesized that the MDA measured in the cell samples may likely originate from the digested “sausage”, not from Caco-2 cells, as studies have reported MDA levels of ca. 0.2 mg/kg in sausages [51]. It has been shown that high-fat-food items can increase peroxide concentrations, causing increased lipid oxidation [52], even during digestion [16]. Whichever the source, MDA can be involved in intracellular adduct formation, such as with cellular DNA, proteins, as well as membranes [52]. MDA production by a Caco-2 co-culture cell model was shown to be reduced by antioxidants such as resveratrol and anti-inflammatory compounds such as eicosapentaenoic acid [53], though such further reductions with antioxidants were not observed in the present study, again possibly indicative of the MDA origin from the food matrix itself.

Although the results gained by the DNA/RNA oxidative damage assay are less pronounced than the results of the FRAP and ABTS tests, an increase in oxidative damage in the cell samples treated with digesta containing “sausage”, “white chocolate”, or their combinations is apparent and support the results obtained from the assays measuring total antioxidant capacity. Surprisingly, unstimulated cells treated with “curcumin” digesta slightly increased DNA/RNA oxidative damage despite increased antioxidant capacities measured during the FRAP and ABTS tests. One possible explanation for this divergence could be the reported DNA damaging effect caused by “curcumin”, showing that, depending on its concentration, curcumin can induce mitochondrial and nuclear DNA damage in a variety of cells, including hepatoma G2 and Caco-2 cells [54,55], again pointing out that exogenous antioxidants may act as double-edged swords.

Regarding the effects of food combinations, the pro-oxidant and pro-inflammatory effects of sausage and white chocolate alone were only reduced slightly, if at all reduced, by the respective combinations with orange juice or coffee. Thus, the combined digesta effects were less than additive and not synergistic in this cellular model, which was different from previous findings when studying effects of the same food items in digesta only. Aspects of limited bioavailability of some of the constituents in the Caco-2 cell model and more complex interactions with this epithelial model may explain these less than additive effect.

The study’s main limitations include studying the effects of digested food items on a simple intestine model constituting only Caco-2 cells. While it is known that this cell line can be stimulated for inflammation [23,32], the epithelium in the small intestine also contains, among others, mucus-producing cells (goblet cells), and immune cells (M-cells, dendritic cells [56]) with the latter likely resulting in a much stronger reaction toward inflammatory stimuli. The mucus layer normally present in the gut could potentially impact the absorption of nutrients and secondary plant compounds, although, in an earlier study, no such reduced cellular uptake was recognized in a cell model containing mucus-producing HT-29-MTX cells [23]. For these reasons, future work could focus on studying the relation of food digestion and the intestinal epithelium in further complex models, potentially even including the large intestine and bacterial responses such as in the dynamic TIM model [57], and further investigate complementary markers of oxidative stress and inflammation, including intracellular signaling cascades such as Nrf-2 and NF-ĸB, and measuring intracellular stress directly [58]. Another possible limitation of the present study is avoiding—similar to previous studies with vegetables [59]—the oral phase of the in vitro digestion. However, based on the INFOGEST model [20], this phase is very short and is more important for starchy foods. In addition, INFOGEST also recommends that, for liquid foods, the oral phase can be circumvented, thus we feel that this is not a strong limitation. The strengths of this study include employing a relevant and differentiated cell line (Caco-2) to simulate the intestinal lining, expressing many transporters also present in the gut [60], and working with digested real food items following a consensus model of digestion and the inclusion of several relevant controls.

## 5. Conclusions

To summarize, the present results confirm our hypothesis and are in line with a previous study on gastro-intestinal digesta [16], namely that food items and combinations presumably rich in pro-oxidants and of possibly pro-inflammatory properties were able to stimulate pro-oxidant and inflammatory related reactions in the gut epithelium following gastro-intestinal digestions, and that food items rich in antioxidants were rather lowering or at least not aggravating induced inflammation and oxidative stress. Further studies are warranted to investigate food-induced oxidative stress and inflammatory aspects, hallmarks for many chronic diseases, in further detail such as elucidating the cellular pathways involved and studying their possible role in systemic inflammation or models thereof.

## Figures and Tables

**Table 1:** The different food items analyzed and their abbreviations used in the study.

Digested Food Items	Amount Digested	Ingredients (from Label), per 100 g or 100 mL	Composition (from Labels)	Abbreviation
Sausage	2 g	Pork liver 25%, pork meat 22%, pork fat, water, pork rind, potato starch, dextrose, salt, cream, sugar, onions, preservatives (potassium acetate, sodium nitrite), spices, emulsifiers (citric acid esters from mono- and diglycerides from fatty acids), antioxidants (ascorbic acid, sodium ascorbate), thickener (xanthan), spice extracts, hemoglobin, aroma	Fat (29 g), saturated fats (11 g), carbohydrates (7.5 g), proteins (9 g), salt (1.8 g), total energy (329 kcal)	M
White chocolate	2 g	Sugar, whole milk powder, cocoa butter, skim milk powder, emulsifier (soya lecithin), flavoring	Fats (25 g), saturated fats (22 g), carbohydrates/of which sugars (55/55 g), proteins (5.7 g), total energy (561 kcal)	W
Soda	2 mL	Sparkling water, sugar, acidifiers: citric acid, malic acid, acidity corrector; sodium gluconate, natural lemon-green lemon aroma, sweetener: steviol glycoside	Fat (0 g), carbohydrates/of which sugars (6.6 g/6.6 g), proteins (0 g), total energy (28 kcal)	S
Orange juice	2 mL	100% juice	Fat (0 g), carbohydrates (8.7 g), proteins (0.7 g), vitamin C (12 mg), total energy (42 kcal)	O
Coffee	2 mL	100% Arabica coffee	ca. 8 mg caffeine *, ca. 100 mg of total polyphenols **	K
Curcumin	12.5 mg	n/a	Curcumin 98%	C
Sausage + Coffee	2 g + 2 mL			MK
Sausage + Orange juice	2 g + 2 mL			MO
White chocolate + Orange juice	2 g + 2 mL			WO
White chocolate + Coffee	2 g + 2 mL			WK
White chocolate + Sausage	2 g + 2 g			WM

* https://fdc.nal.usda.gov/fdc-app.html#/food-details/171890/nutrients (accessed on 30 January 2023). ** http://phenol-explorer.eu/contents/food/662 (accessed on 30 January 2023); [19].

**Table 2:** Treatment of different Caco-2 exposure groups.

Exposure Group	Abbreviation	Treatment of Caco-2 Cells ^d^
Blank (medium)	med.	Untreated: no stimuli or digesta
Blank + stimuli	med.st.	No digesta, stimulation with TNF-α, IL1β, LPS, H_2_O_2_ (20 h) ^a^
Blank + stimuli + empty digesta ^b^	med.st.e.dig.	Stimulation with TNF-α, IL1β, LPS, H_2_O_2_ (20 h) and empty digesta (hours 4–20)
Blank + stimuli + digesta 1–11 ^c^	digesta.st.	Stimulation with TNF-α, IL1β, LPS, H_2_O_2_ (20 h) and digesta 1–11 (hours 4–20)
Blank + digesta 1–11	digesta	No stimulation, digesta 1–11 (hours 4–20)
Blank + empty digesta ^b^	med.e.dig	No stimulation, empty digesta (hours 4–20)

^a^ 100 ng mL^−1^ TNF-α, 25 ng mL^−1^ IL1β, 10 µg mL^−1^ LPS, and 0.4 mmol L^−1^ H_2_O_2_. ^b^ equals control. ^c^ digesta with food matrices (see Table 1). ^d^ n = 1; N = 3 per treatment: n = technical replicate, same day experiment; N = considered biological replicate, experiment from a different day.

## Data Availability

Data are contained within the article or Supplementary Material.

## Supplementary Materials

The following supporting information can be downloaded at: https://www.mdpi.com/article/10.3390/antiox13020150/s1, Table S1: Standard dilution series for F2-isoprostane analysis; Table S2: Standard dilutions for the ABTS assay; Table S3: Standard dilutions for the FRAP assay; Table S4: Standard dilutions for the IL-6 assay; Table S5: Standard dilutions for the MDA assay; Table S6: Standard dilutions for DNA/RNA oxidative damage assay.
