# Supplementary material for: Effect of Digested Selected Food Items on Markers of Oxidative Stress and Inflammation in a Caco-2-Based Human Gut Epithelial Model
Source: Antioxidants (Basel). 2024 Jan 25;13(2):150. doi: 10.3390/antiox13020150 (PMC10885899; doi:10.3390/antiox13020150)
Supplement: Supplementary file 1 [file antioxidants-13-00150-s001.zip › antioxidants-2802263-supplementary.pdf]

Supplementary Table 1. Standard dilution series for F2- isoprostane analysis.

Number	V (ELISA buffer) (µL)	V (standard) (µL)	concentration (pg/mL)
**1**	900	100 (5 ng/ml)	500
**2**	750	500 std 1	200
**3**	750	500 std 2	80
**4**	750	500 std 3	32
**5**	750	500 std 4	12.8
**6**	750	500 std 5	5.1
**7**	750	500 std 6	2
**8**	750	500 std 7	0.8

Supplementary Table 2. Standard dilutions for the ABTS assay. Starting standard concentration was 10 mg/mL.

Standards	Dilution	Volume ascorbic acid stock solution (µL)	Volume deionized H_2O (µL)	Concentration (mg/mL)
**1**	/	/	1000	0
**2**	1:200	5	995	0,05
**3**	1:100	10	990	0,1
**4**	1:50	20	980	0,2
**5**	1:33,33	30	970	0,3
**6**	1:25	40	960	0,4
**7**	1:20	50	950	0,5

Supplementary Table 3. Standard dilutions for the FRAP assay. Starting concentration was 2535 mg/L.

Standard	Dilution	Volume stock (µL)	Distilled water (µL)	Concentration (mg/L)
**Blank**	/	0	1000	0
**Standard 1**	1:400	2.5	997.5	6.34
**Standard 2**	1:200	5	995	12.68
**Standard 3**	1:133.33	7.5	992.5	19.02
**Standard 4**	1:100	10	990	25.35
**Standard 5**	1:66.66	15	985	38.03
**Standard 6**	1:50	20	980	50.7
**Standard 7**	1:33.33	30	970	76.06
**Standard 8**	1:25	40	960	101.44
**Standard 9**	1:20	50	950	126.8

Supplementary Table 4a. Standard dilutions for the IL-6 assay.

Number	V(ELISA assay buffer) (µl)	V (standard) (µL)	IL-6 concentration (pg/mL)
1	**350 (for 3 blanks)**	**0**	**0**
2	475	25 (5000 pg/mL)	250
3	250	250 std 2	125
4	250	250 std 3	62,5
5	250	250 std 4	31,2
6	250	250 std 5	15,6
7	250	250 std 6	7,8
8	250	250 std 7	3,9

Supplementary Table 4b. Standard dilutions for the IL-8 assay.

Number	V(ELISA/ELISPOT diluent) (μΛ)	V (standard) (µl)	IL8 concentration (pg/mL)
1	0	120 stock	250
2	120	120 µL std 1	125
3	120	120 µL std 2	62.5
4	120	120 µL std 3	31.2
5	120	120 µL std 4	15.6
6	120	120 µL std 5	7.8
7	120	120 µL std 6	3.9
Blank	120	0	0

Supplementary Table 5. Standard dilutions for the MDA assay.

Tube	MDA 25 µM (µL)	Water (µL)	MDA concentration (µM)
**A**	0	500	0
**B**	2.5	498	0.125
**C**	5	495	0.25
**D**	10	490	0.5
**E**	20	480	1
**F**	50	450	2.5
**G**	100	400	5
**H**	200	300	10
**I**	400	100	20

Supplementary Table 6. Standard dilutions for DNA/RNA oxidative damage assay.

Number	V(ELISA Buffer) (µL)	V (standard) (µL)	concentration (pg/mL)
**1**	900	100 (30 ng/mL)	3000
**2**	500	400 std 1	1333
**3**	500	400 std 2	592.6
**4**	500	400 std 3	263.4
**5**	500	400 std 4	117.1
**6**	500	400 std 5	52
**7**	500	400 std 6	23.1
**8**	500	400 std 7	10.3
